# Molecular Determinants of Filament Capping Proteins Required for the Formation of Functional Flagella in Gram-Negative Bacteria

**DOI:** 10.3390/biom11101397

**Published:** 2021-09-22

**Authors:** Marko Nedeljković, Sandra Postel, Brian G. Pierce, Eric J. Sundberg

**Affiliations:** 1 Department of Biochemistry, Emory University School of Medicine, Atlanta, GA 30322, USA; marko.nedeljkovic@emory.edu; 2 Institute of Human Virology, University of Maryland School of Medicine, Baltimore, MD 21201, USA; s.postel@gmx.de; 3 Institute for Bioscience and Biotechnology Research, University of Maryland, Rockville, MD 20850, USA; pierce@umd.edu; 4 Department of Cell Biology and Molecular Genetics, University of Maryland, College Park, MD 20742, USA

**Keywords:** bacterial flagella, FliD, filament cap, *Pseudomonas aeruginosa*

## Abstract

Bacterial flagella are cell surface protein appendages that are critical for motility and pathogenesis. Flagellar filaments are tubular structures constructed from thousands of copies of the protein flagellin, or FliC, arranged in helical fashion. Individual unfolded FliC subunits traverse the filament pore and are folded and sorted into place with the assistance of the flagellar capping protein complex, an oligomer of the FliD protein. The FliD filament cap is a stool-like structure, with its D2 and D3 domains forming a flat head region, and its D1 domain leg-like structures extending perpendicularly from the head towards the inner core of the filament. Here, using an approach combining bacterial genetics, motility assays, electron microscopy and molecular modeling, we define, in numerous Gram-negative bacteria, which regions of FliD are critical for interaction with FliC subunits and result in the formation of functional flagella. Our data indicate that the D1 domain of FliD is its sole functionally important domain, and that its flexible coiled coil region comprised of helices at its extreme N- and C-termini controls compatibility with the FliC filament. FliD sequences from different bacterial species in the head region are well tolerated. Additionally, head domains can be replaced by small peptides and larger head domains from different species and still produce functional flagella.

## 1. Introduction

Pathogenic bacteria that cause a multitude of diseases in humans often rely on flagella-based motility through liquid environments to reach and adhere to host target cells, making bacterial flagella nearly indispensable for effective infection and colonization [1,2]. A membrane-localized motor base drives the propeller-like motion of a connected extracellular filament. The flagellar filament is composed of thousands of copies of the protein flagellin, or FliC, ordered in a helical manner around a central pore with a capping protein complex composed of FliD protein subunits that form a chamber at the distal end of the filament. Individual unfolded subunits of FliC traverse through the hollow filament pore from the bacterial cytoplasm prior to being folded and sorted into position at the tip of the growing filament [3]. The exact role of the FliD cap in FliC folding and sorting is not fully understood. However, that some proteins are permitted to be secreted into the medium suggests that it has a more active role than as just a plug that prevents the leakage of FliC, possibly acting like an Anfinsen cage [4,5]. In bacteria that do not express FliD, flagellar filaments are not formed, resulting in impaired motility and infectivity. In the assembled filament, flagellin subunits switch between two different states in order to supercoil, generating “short” and “long” protofilaments, allowing the rotating filament to act as an Archimedean screw that produces the thrust that drives swimming motility [6,7].

FliD capping protein complexes have been shown to adopt distinct oligomeric states in different bacteria—hexamers in *Pseudomonas aeruginosa* and *Escherichia coli* [8,9], pentamers in *Salmonella* and *Campylobacter jejuni* [4,9,10,11,12] and tetramers in *Serratia marcescens* and *Bdellovibrio bacteriovorus* [13,14]. FliD proteins from *Salmonella* and *Pseudomonas* are not interchangeable, such that the expression of the *fliD* gene from *Salmonella* in a *Pseudomonas ΔfliD* knockout strain does not restore bacterial motility and vice versa [15]. This is likewise the case for *Pseudomonas* and *E. coli*—swapping *fliD* genes from these bacteria does not impart motility, despite FliD proteins from both of these bacteria forming hexamers. Conversely, both FliD and FliC from *E. coli* and *Salmonella* are functionally interchangeable in these bacteria [15], even though their FliD proteins can assemble into filament-capping complexes with distinct oligomeric states, at least in the bacteria from which they are derived. Near-atomic resolution cryo-electron microscope (cryo-EM) analyses of filaments from several different species clearly show that all studied bacteria produce filaments comprised of eleven protofilaments with conformational similarity in the inner domains and structural diversity in the outer domains [16,17,18,19]; no such structure of the *E. coli* flagellar filament exists at any resolution. Thus, with 11-mer FliC filaments capped by either pentameric or hexameric FliD complexes, there is no obvious correspondence between oligomeric states of FliC filaments and FliD capping protein complexes that confers functional flagella in bacteria.

FliD is highly flexible [8,20], a property that is thought to facilitate its ability to engage the FliC molecule exiting the distal end of the filament, direct its folding and position it such that the filament can grow. However, little is known about the molecular mechanisms by which FliD accomplishes each of these steps in flagellar formation. Structural studies [9] have led to a model in which symmetry mismatches (i.e., the number of FliC protofilaments is not an integer multiple of the number of subunits in the FliD capping protein complex) necessitate a distortion of the regular polygonal shape, whether pentagonal or hexagonal, of the capping protein complex. This results in each FliD subunit occupying a position between FliC protofilaments, which creates mechanical strain that ratchets the capping protein complex in the direction of filament growth as each new FliC protein emerges, folds and is positioned. Regardless of the exact molecular mechanism by which FliC subunits are folded into the growing flagellar filament, any model of filament assembly would require direct but transient interactions between FliD and FliC proteins.

In order to define which parts of FliD proteins are critical for its function and FliD-FliC compatibility (i.e., recognition of the species-specific FliC) in *Salmonella*, *Pseudomonas* and *E. coli*, we employed complementary methods in bacterial genetics, motility assays and molecular modeling. Our data provide novel insights into how FliD could function as a chaperone for FliC folding and filament assembly at the distal end of bacterial flagella, implicating a flexible coiled coil formed by the extreme N- and C-termini as the only region of FliD that comprises functional determinants that are intolerant to substitution, whereas other FliD regions can undergo extensive deletion, mutation and/or substitution.

## 2. Results

### 2.1. Design of FliD Truncations and Chimeras

**Figure 1:**
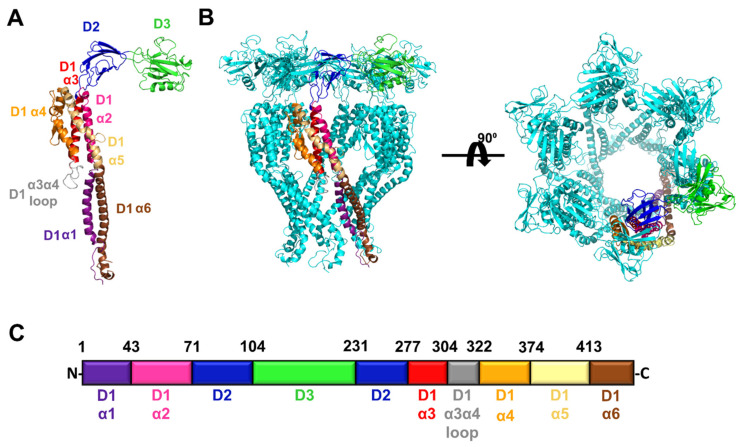
Structural model of full length FliD from the *P. aeruginosa* PAO1 strain. (**A**) Model of the FliD monomer. The α helices in the D1 domain are individually colored and labeled; domains D2 and D3 are individually colored and labeled. (**B**) Model of the FliD hexamer. A single FliD monomer is colored as in (**A**); the remaining five monomers in the hexamer are in cyan. (**C**) Organization of the *P. aeruginosa* PAO1 FliD protein. Colors of domains or secondary structural elements within domains correspond to those in (**A**). The N- and C-termini, as well as the amino acid positions at the boundaries between domains or secondary structural elements, are indicated.

In order to determine which regions of FliD flagellar capping proteins are required for function and cross-species compatibility with the flagellar filament, we first modeled the full-length FliD protein from the *P. aeruginosa* PAO1 strain using the I-TASSER server [21], followed by refinement in Rosetta [22]. The flexibility of FliD has prohibited the high-resolution visualization of the entire protein—in the first X-ray crystal structure of a FliD protein, from *Pseudomonas*, only the two head/plate domains (the D2 and D3 domains) and a single helix (α3) from the helical bundle domain (D1) exhibited sufficient electron density to reliably build the structure despite a larger fragment that included the entire protein sequence except the first, second and final α helices of the D1 domain having been crystallized [8]. A more recent structure of the *E. coli* FliD protein includes all but the first and last α helices (α1 and α6) of the D1 domain [9]. Both *Pseudomonas* and *E. coli* FliD proteins self-organize into hexameric complexes [8,9] and, thus, we used these structures to model the full-length *Pseudomonas* FliD structure in both monomeric (Figure 1A) and hexameric (Figure 1B) forms. Our models indicate that the first (D1-α1) and final (D1-α6) helices in the D1 domain likely form a coiled coil that is highly mobile (discussed below). In total, *Pseudomonas* FliD is constructed from three domains, two of which (D2 and D3) are predominantly composed of β strands and form the head/plate region, and one of which (D1) is entirely helical with six α helices and forms the leg regions. Representative of all FliD proteins, the domain architecture of *Pseudomonas* FliD is constructed from a discontinuous sequence—the head/plate region is formed from residues located in sequence between the D1 α2 and α3 helices and the D3 domain is a loop insertion of the D2 domain (Figure 1C and Supplemental Figure S1). In the experiments described below, we used this full-length FliD model to design all truncated and chimeric genes in which we have either removed amino acids corresponding to individual D1 α helices, entire domains or combinations thereof, or replaced them with analogous parts of FliD from other bacteria.

### 2.2. FliD Head/Plate Regions Are Interchangeable between Diverse Bacteria

**Figure 2:**
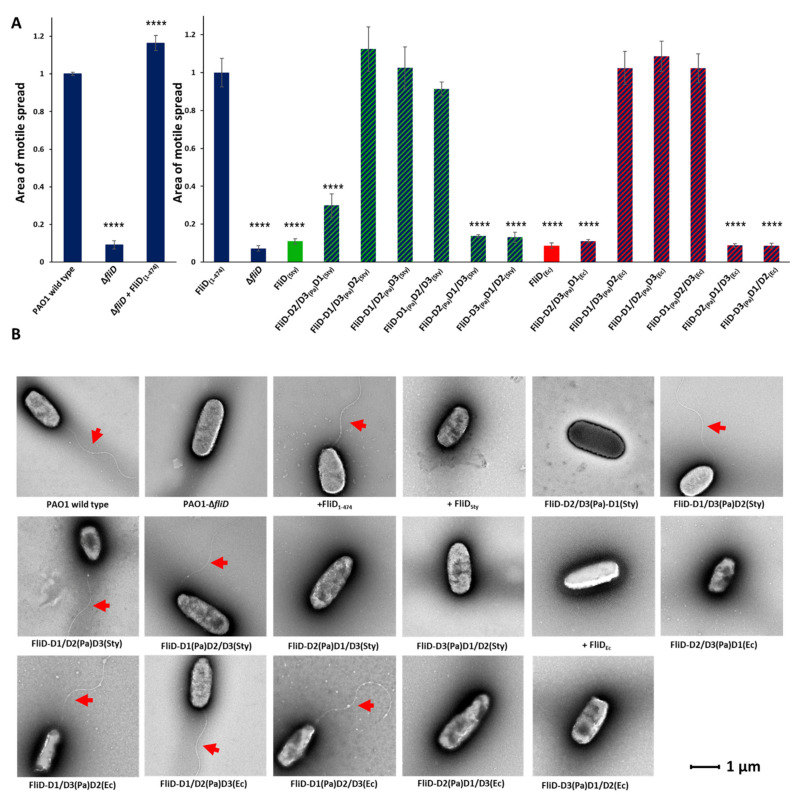
Effects of FliD head/plate domain swaps in flagellar formation and function. (**A**) Swimming motility analysis and (**B**) negative-stain EM images of knockout and complemented *P. aeruginosa* PAO1 strains. Origin of each domain (D1, leg domain; D2, first head/plate domain; D3, second head/plate domain) in the chimeric *fliD* gene in each complement is indicated. Area of motile spread for each strain is normalized to that of the full-length wild type complemented strain [*Pa*Δ*fliD*/*fliD_Pa_*(1-474)]. (**** *p* ˂ 0.0001).

Previously, only one study addressed the exchangeability of FliD proteins among different species [15], showing that full-length FliD molecules are interchangeable between *Salmonella* and *E. coli*, but not between any of the two species and *Pseudomonas*. Thus, we sought to determine which parts of FliD were interchangeable between FliD proteins from diverse bacteria. We constructed FliD chimeras from different bacteria to define the regions of FliD that are necessary for cross-bacterial compatibility with the FliC filament and, thereby, swimming motility function. To investigate which parts of FliD are involved in flagellar assembly, we cloned multiple *fliD* chimeric genes in which we swapped head/plate domain 2 [e.g., *fliD_Pa_*(D1/D3)*fliD_Sty_*(D2); *fliD_Pa_*(D1/D3) *fliD_Ec_*(D2)], head/plate domain 3 [e.g., *fliD_Pa_*(D1/D2)*fliD_Sty_*(D3); *fliD_Pa_*(D1/D2)*fliD_Ec_*(D3)] regions or both head/plate domains [e.g., *fliD_Pa_*(D1)*fliD_Sty_*(D2/D3); *fliD_Pa_*(D1)*fliD_Ec_*(D2/D3)] from either *Salmonella* or *E. coli* into the *fliD* gene from *Pseudomonas* and vice versa [e.g., *fliD_Pa_*(D2/D3)*fliD_Sty_*(D1)] and complemented the *fliD* transposon strain PW2975 (a *Pseudomonas* Δ*fliD* strain) with a plasmid-borne wild type or chimeric *fliD* genes. All chimeric genes were codon-optimized for expression in the *P. aeruginosa* PAO1 strain. We then tested all of the resulting bacterial strains expressing chimeric FliD proteins for their ability to restore swimming motility in *Pseudomonas* and directly observed flagella by negative-stain electron microscopy (EM). The Δ*fliD* strain exhibits severely impaired swimming motility and flagellar formation (Figure 2 and Supplemental Figure S2), both of which are restored by complementation with the full-length wild type *fliD* gene in the *Pa*Δ*fliD/fliD_Pa_*(1-474) strain. We observed that the *Pseudomonas* FliD head/plate domains, D2 and D3, could be replaced individually or together with the analogous FliD domains from either *Salmonella* or *E. coli* with no effect on swimming motility, whereas replacement with entire FliD proteins from either *Salmonella* or *E. coli* reduced swimming motility to levels indistinguishable from the *Pa*Δ*fliD* strain (Figure 2). When we constructed the reverse chimeras in which all FliD parts except for the head/plate domains, D2 or D3, either individually or together, were derived from either *Salmonella* or *E. coli*, we observed abolished swimming motility in *Pseudomonas* (Figure 2).

These data indicate that only the FliD head/plate domains are completely interchangeable between the three species tested, but that not all parts of FliD outside of the head domains exhibit cross-bacterial compatibility. In each chimeric *fliD* strain in which swimming motility was restored to wild type levels, we observed flagella by negative-stain EM that exhibited the appearance of wild type flagella; in all of the chimeric *fliD* strains in which swimming motility was abolished, we observed no flagella (Figure 2).

### 2.3. Flagellar Filament Compatibility Depends Solely on the FliD N- and C-Terminal Helices

**Figure 3:**
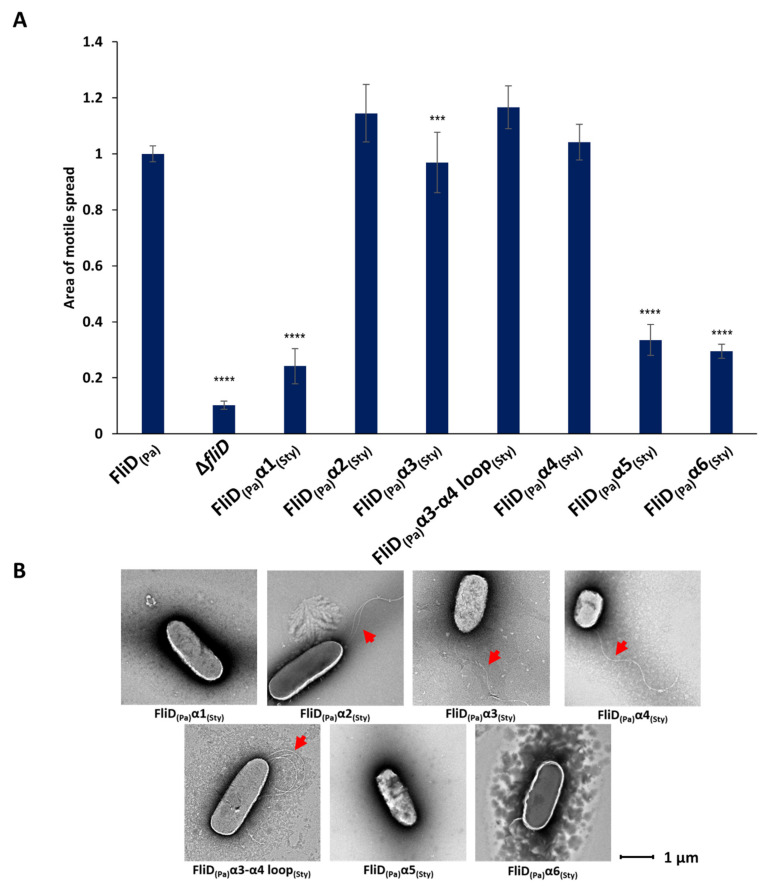
Effects of FliD D1 helical swaps in flagellar formation and function. (**A**) Swimming motility analysis and (**B**) negative-stain EM images of knockout and complemented *Pseudomonas aeruginosa* PAO1 strains. Origin of each helix (α1, α2, α3, α3-α4 loop, α4, α5, α6) in the chimeric *fliD* gene in each complement is indicated. Area of motile spread for each strain is normalized to that of the full-length wild type complemented strain [*Pa*Δ*fliD*/*fliD_Pa_*(1-474)]. (*** *p* ˂ 0.001; **** *p* ˂ 0.0001).

The above-described chimera studies indicated that all or part of the FliD leg (D1) domain dictates compatibility with the flagellar filament. FliD leg domains are each composed of six α helices—α1 and α2, which reside N-terminal, and α3 through α6, which reside C-terminal, to the head/plate (D2 and D3) domains, which collectively form a single helical bundle structure. In order to determine which α helices in the leg domain are responsible for functional compatibility with the flagellar filament, we constructed additional chimeric bacterial strains in which we replaced each of the six individual α helices in *Pseudomonas* FliD with the analogous helices from *Salmonella* FliD and assessed the swimming motility of each of the resulting strains. We found that only chimeric strains that contained the α1, α5 or α6 helices belonging to *Salmonella* [*Pa*Δ*fliD/fliD_Sty_*(α1), *Pa*Δ*fliD/fliD_Sty_*(α5) and *Pa*Δ*fliD/fliD_Sty_*(α6), respectively] exhibited significantly impaired swimming motility (Figure 3 and Supplemental Figure S3). Conversely, chimeras containing the α2, α3 or α4 helices, or the loop between the α3 and α4 helices, of *Salmonella* [*Pa*Δ*fliD/fliD_Sty_*(α2), *Pa*Δ*fliD/fliD_Sty_*(α3) and *Pa*Δ*fliD/fliD_Sty_*(α4), *Pa*Δ*fliD/fliD_Sty_*(α3-α4 loop), respectively] were functionally tolerated, exhibiting swimming motility equivalent to the *Pseudomonas* wild type *fliD* complemented strain, *Pa*Δ*fliD*/*fliD_Pa_*(1-474) (Figure 3). As we had observed for other chimeric strains, those that had impaired swimming motility formed no flagella, while those that had wild type-level swimming motility formed flagella, as determined by negative stain EM (Figure 3). In our model of full-length *Pseudomonas* FliD (Figure 1), the N-terminal α1 and C-terminal α6 helices form a coiled coil, a feature already suggested in previous FliD sequence analysis [20] and further corroborated by a coiled coil prediction tool DeepCoil [23]. The α5 helix is nearly contiguous with the α6 helix, whereas the α2, α3 and α4 helices bundle together proximal to the head/plate domains. These data indicate that the molecular determinants of functional compatibility between the FliD capping protein complex and the FliC filament reside solely in a discrete coiled coil region extending from the core helical bundle of the FliD leg domain.

### 2.4. D1 Domain Is Essential for Flagellar Formation and Swimming Motility

**Figure 4:**
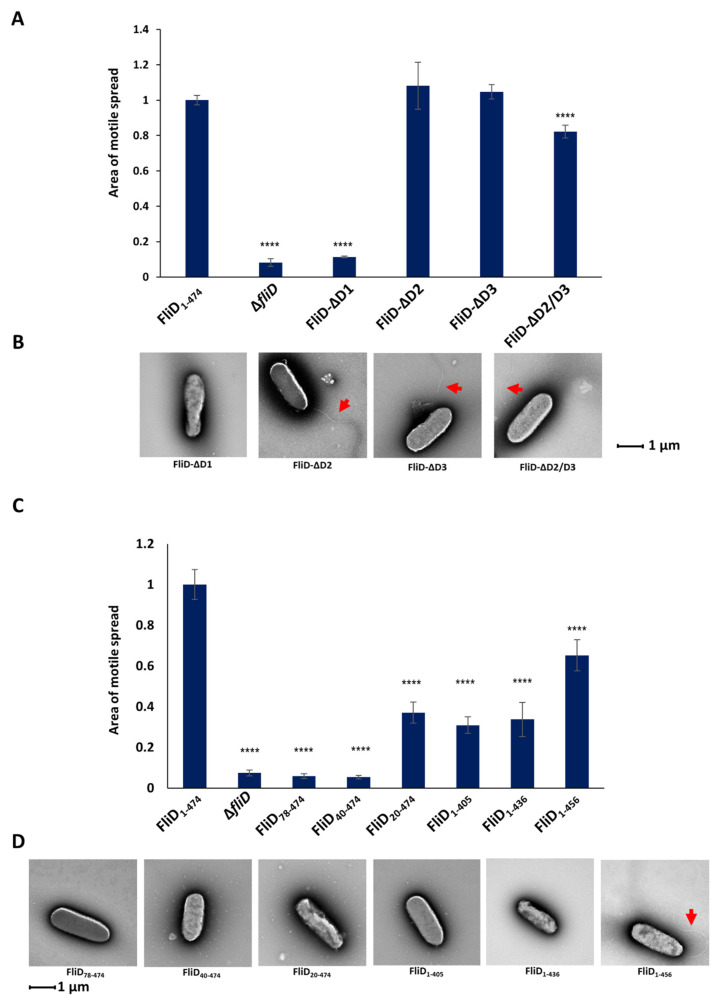
Effects of FliD truncations in flagellar formation and function. Swimming motility analysis and negative-stain EM images of knockout and complemented *P. aeruginosa* PAO1 strains of domain deletions (**A**,**B**) and domain D1 truncations (**C**,**D**). Area of motile spread for each strain is normalized to that of the full-length wild type complemented strain [*Pa*Δ*fliD*/*fliD_Pa_*(1-474)]. (**** *p* ˂ 0.0001).

In order to determine the contribution of individual domains of the *Pseudomonas* FliD to its function, we generated *fliD* genes in which D1, D2, D3 or both D2 and D3 domains were deleted, and assessed for filament formation and swimming motility. We found that the removal of the entire D1 domain made cells completely immotile, with no observable flagella (Figure 4 and Supplemental Figure S4). Conversely, deletion of either D2 or D3 resulted in fully formed flagella and had no effect on their motility, while deletion of both of these domains led to a small but statistically significant decrease in motile spread compared to the wild type. Next, we assessed whether less severe truncations of D1 would also impair swimming motility and flagella formation. We tested truncations from both the N- and C- termini [*Pa*Δ*fliD/fliD_Pa_*(78-405)—removal of the N-terminal 77 residues up to the head/plate region, as well as the C-terminal 69 residues], the N-terminus alone [*Pa*Δ*fliD/fliD_Pa_*(78-474)—removal of the D1 domain sequence prior to the head/plate region; *Pa*Δ*fliD/fliD_Pa_*(40-474)—removal of the N-terminal 39 residues and *Pa*Δ*fliD/fliD_Pa_*(20-474)—removal of the N-terminal 19 residues] and the C-terminus alone [*Pa*Δ*fliD/fliD_Pa_*(1-405)—removal of the C-terminal 69 residues; *Pa*Δ*fliD/fliD_Pa_*(1-436)—removal of the C-terminal 38 residues; *Pa*Δ*fliD/fliD_Pa_*(1-456)—removal of the C-terminal 18 residues]. For each of these strains complemented with truncated *fliD* genes, we observed a statistically significant reduction in swimming motility. For *Pa*Δ*fliD/fliD_Pa_*(78-474) and *Pa*Δ*fliD/fliD_Pa_*(40-474) the reduction was similar to that of the Δ*fliD* strain. We found no visual evidence for the formation of flagella extending from the bacteria surfaces, with the exception of *Pa*Δ*fliD/fliD_Pa_*(1-456) (Figure 4). Thus, removal of any part of the FliD protein in *Pseudomonas* bacteria, including regions as short as 18 residues, from either of its termini significantly impairs swimming motility.

### 2.5. Head/Plate Domains Can Be Replaced by an Unrelated Protein Domain

**Figure 5:**
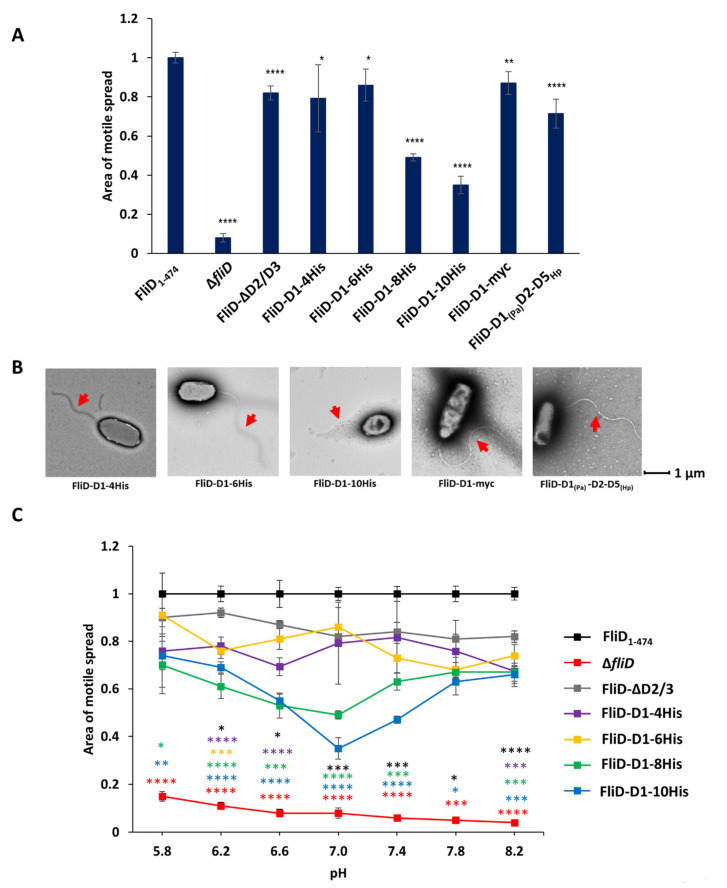
Effects of head/plate domains replacement in flagellar formation and function. Swimming motility analysis (**A**) and negative-stain EM images (**B**) of knockout and complemented *P. aeruginosa* PAO1 strains. Head/plate domains were replaced by a peptide tag or equivalent domains from *Helicobacter pylori*. (**C**) The effect of pH on motile spread of PAO1 strains complemented by *fliD*-ΔD2/3 containing polyhistidine tags of different length. Area of motile spread for each strain is normalized to that of the full-length wild type complemented strain [*Pa*Δ*fliD*/*fliD_Pa_*(1-474)]. Motile spread of different strains compared to that of FliD-ΔD2/3 strain. (* *p* ˂ 0.05; ** *p* ˂ 0.01; *** *p* ˂ 0.001 **** *p* ˂ 0.0001).

Our results demonstrate that the functional and species compatibility determinants are located on the D1 domain of FliD and that the head D2 and D3 domains are entirely dispensable for its function. We next wanted to determine how replacement of D2 and D3 domains by a foreign peptide would affect the formation and function of the filament. To test this, we constructed chimeric genes in which we replaced head/plate domains by a linker consisting of an increasing number of histidine residues between four and ten (*fliD_Pa_-D1-4his, fliD_Pa_-D1-6his, fliD_Pa_-D1-8his* and *fliD_Pa_-D1-10his*). We then complemented the *PaΔfliD* strain with these chimeras and assessed filament formation and swimming motility in the conditions used in all experiments described above. For the strains complemented with *fliD_Pa_-D1-4his* and *fliD_Pa_-D1-6his*, motile spread was indistinguishable from that of the FliD-ΔD2/D3 (Figure 5A and Supplemental Figure S5). Conversely, strains complemented with *fliD_Pa_-D1-8his* and *fliD_Pa_-D1-10his* exhibited significantly decreased motility in agar, with the latter having an average spread of only one-third of the full-length complement. Moreover, the filaments of *PaΔfliD/fliD_Pa_-D1-10his* appeared to be significantly shorter compared to those of the *PaΔfliD/fliD*-ΔD2/D3 complement (Figure 5B). To determine whether the length of the insert *fliD_Pa_-D1-10his* was the cause of this effect, we constructed additional genes in which the head regions of *Pseudomonas* were replaced by a 10-residue long Myc tag (*fliD_Pa_-D1-myc*) or the head region from the *Helicobacter pylori* FliD, (*fliD_Pa_*(D1)*fliD_Hp_*(D2-D5), which has four domains and is twice the size of FliD from *Pseudomonas*. Our assays showed that motility of *PaΔfliD/fliD_Pa_-D1-myc* and of *PaΔfliD/fliD_Pa_*(D1)*fliD_Hp_*(D2-D5) were comparable to of *PaΔfliD/fliD_Pa_*-ΔD2/D3, indicating that the size of the replacement domain was not the limiting factor for the formation of functional filaments. What is common for the *Pseudomonas* and *H. pylori* FliD head domains, as well as the Myc tag chimera, is that their isoelectric points are below five, and these assays were performed in agar media with phosphate buffer to maintain neutral pH 7 during the experiment. Since the isoelectric point of the 10His tag is 7.3, we examined whether its negative effect on motility was due to the pH of the environment. We tested the swimming of the full-length complement, as well as *PaΔfliD/fliD_Pa_-D1*, *PaΔfliD/fliD_Pa_-D1-4his*, *PaΔfliD/fliD_Pa_-D1-6his*, *PaΔfliD/fliD_Pa_-D1-8his* and *PaΔfliD/fliD_Pa_-D1-10his* across a pH range of pH 5.8 and 8.2. Our results showed that the pH of the media had a statistically significant effect on *PaΔfliD/fliD_Pa_-D1-8his* and *PaΔfliD/fliD_Pa_-D1-10his*, the motility of which significantly decreased when the pH of the media was close to the isoelectric points of the inserts, with stronger effect on *PaΔfliD/fliD_Pa_-D1-10his* than on *PaΔfliD/fliD_Pa_-D1-8his* (Figure 5C). Conversely, this effect was not observed for the other complemented strains we tested, suggesting that the combination of the insert length and physicochemical properties of histidine together affected the function of D1 and, consequently, swimming motility.

### 2.6. Determinants of Functional Compatibility in FliD Are Exceptionally Flexible

**Figure 6:**
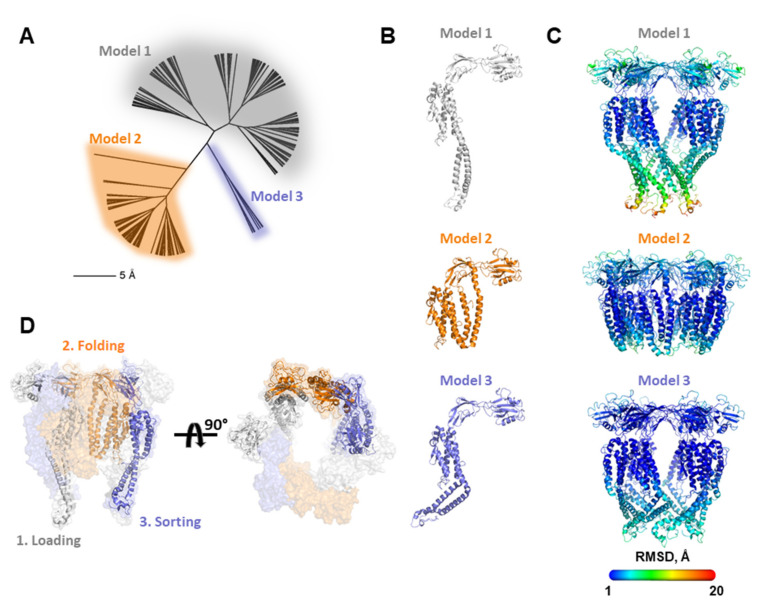
Molecular modeling of FliD conformations. (**A**) Conformational cluster analysis of FliD molecular models indicating three major clusters of conformationally distinct models; (**B**) representative FliD monomer structures from each of the three clusters in (**A**); (**C**) representative FliD hexamer structures from each of the three clusters in (**A**). Structures are colored according to heat map of per residue RMSD between all models from 1 to 20 Å (*legend*). (**D**) Multiple FliD conformations by individual FliD subunits can be accommodated in the same FliD capping protein complex as it cycles through steps of loading, chaperoning and sorting individual FliC subunits. Only the three FliD subunits involved in these steps are shown as cartoon renderings.

The process of flagellar formation is a highly dynamic process in which each FliC must travel in an unfolded, or partially folded, state through the 25 Å inner diameter filament pore to its end where it folds against, and is sorted, by the FliD capping protein complex. Models of filament growth [9,11] suggest that FliD monomers within the oligomeric complex adopt distinct conformations depending on which position they occupy relative to the location of the FliC subunit that is at that moment being folded and sorted into its final position in the flagellar filament. No structural data are yet available showing these different FliD conformations at any resolution and it remains unclear whether all FliD regions are equally flexible, or some regions are more flexible while others are more rigid. Thus, in order to better understand the conformational flexibility inherent in FliD proteins, we used molecular modeling to determine discrete low energy conformations that the full-length *Pseudomonas* FliD protein may adopt. After generating a full-length model of FliD by I-TASSER [21], we used Rosetta’s FloppyTail application [24] to perform fully flexible modeling of the *Pseudomonas* FliD termini. The application produced 10,000 FliD models, which we clustered (Figure 6A) to select three representative low-energy FliD monomer models (Figure 6B). We assembled each of the three representative monomer models into a distinct hexameric structure based on the FliD_78-405_ crystallographic structure [8], followed by constrained minimization in Rosetta to remove minor clashes. We assessed potential conformational flexibility in these hexameric models using the unconstrained FastRelax protocol in Rosetta (Figure 6C). We found that the majority of the FliD protein, including all of the head/plate domains and most of the leg domain (except some loop regions), is rigid, with very little conformational variability in those regions in our models. Conversely, we observed numerous conformations for the D1 domain α1 and α6 helices. The three low-energy FliD models suggest that the α1 and α6 helices adopt and maintain a coiled coil structure that hinges about a point at the α1/α2 and α5/α6 boundaries in the FliD capping protein complex (Figure 6B,C). Notably, these conformations encompass positions ranging from partially occluding the orifice at the bottom of the FliD capping protein complex to the top of head/plate domains. These modeling data suggest that FliD proteins can adopt a sufficiently extended range of conformations to span regions of the growing flagellum as distant as the pore at the distal end of the FliC filament to the extreme distal surface of the FliD head/plate regions.

## 3. Discussion

The formation of bacterial flagellar filaments is one of the most dynamic processes in biology. In a highly orchestrated series of transient interactions, conformational changes and molecular movements, each of thousands of individual FliC subunits travels from the cytoplasm of the bacterium through the flagellar pore in an unfolded or partially unfolded state, engages the FliD cap assembly oligomer, folds into its native and active conformation, and occupies its proper position in the growing filament. All the while, the distance between the filament pore entrance and exit is increasing as new FliC subunits are placed into position. The FliD cap assembly oligomer is, likewise, a highly dynamic and active participant in these events. As the filament grows, the FliD oligomer corkscrews away from the bacterial cell wall and the leg regions of the individual FliD monomers within the cap assembly adopt different conformations in order to accommodate each extruding FliC subunit, facilitating its folding and placement

A spate of recent high-resolution structures of FliD cap assembly oligomers [8,9,13,25], of varying degrees of completeness, have provided new insights into how FliD functions in its critical and myriad roles in filament assembly. However, these static snapshots of FliD in a single low-energy state are insufficient to explain the spectrum of conformational dynamics undertaken by FliD and to provide a molecular mechanism for filament formation that approaches the level required for rationalizing the development of flagella-specific modulators that could inhibit bacterial motility and infectivity. Appreciating the limits of current structural biology methodologies, we turned to a combination of bacterial genetics, functional assays with live bacteria and molecular modeling in order to better define the molecular determinants of FliD that are functionally required for the intricate molecular choreography in which FliC and FliD proteins participate.

Taking advantage of the incompatibility of FliC and FliD proteins from diverse bacteria, especially but not exclusively between *Pseudomonas* and *Salmonella* bacteria, we engineered a series of chimeric *fliD* genes with which we were able to define the molecular determinants in FliD proteins that are required for functional filament formation. Our experiments indicate that only the extreme N- and C-terminal helices, inclusive of the α1, α5 and α6 helices in the D1 or leg domain, are required for inter-bacterial compatibility. Our data suggest that these structural elements in FliD are used to interact specifically with the individual FliC subunits during the process in which they appear from the distal end of the filament, are folded into their active conformation and placed into their proper position in the growing filament. That the extreme C-terminal helix, α6, of FliD also specifically engages its chaperone inside the bacterium, FliT [26,27], to induce a binding-competent conformation that allows interaction with the flagellar export gate platform protein FlhA [28], further suggests that this part of FliD constitutes a hot spot for interaction throughout the flagellar assembly process. The importance of the terminal region of FliD was further confirmed in our truncation experiments, in which all but one truncated version of FliD failed to produce functional filaments. N-terminal regions of flagellar proteins that are exported through the flagellar type III secretion system also serve as secretion signals [29], which could explain why N-terminal truncations have more profound effect on the phenotype.

Although no X-ray crystal structures of any FliD from any bacterium include sufficient electron density in which to build the N- and C-terminal helices, α1 and α6, respectively, our molecular modeling data suggest that these helices form a coiled coil that can pivot such that the termini can reach from a fully extended position towards the distal end of the filament all the way to the top of the head/plate region. Conversely, the remaining structural elements, including other parts of the leg domain and the entirety of the head/plate domains, appear to be relatively rigid.

The degree of involvement of FliD in the filament formation is still a matter of debate. The simplest explanation suggests that the FliD cap serves as a plug at the distal end of the filament, preventing the leakage of FliC. The strains in which the *fliD* gene was deleted do not form flagellar filaments and are completely immotile. In the early work on *Salmonella* hook-associated proteins, it was shown that the motility in Salmonella *ΔfliD* strains can be rescued in vitro by adding a high concentration of exogenous FliC [30,31]. While these results could lead to the assumption that the *ΔfliD* cells might recover motility in sufficiently dense cultures in which medium becomes crowded with FliC over time, we could not observe such phenomena in *P. aeruginosa* PAO1 and *S. typhimurium ΔfliD* cells, neither in dense liquid cultures nor in agar plates. This, together with the study showing that the native capped filament is significantly more resistant to proteolysis and temperature-induced depolymerization compared to the uncapped or in vitro capped filaments [32] suggests that in native conditions FliD is indispensable for a proper filament synthesis.

The chaperoning role for the FliD cap was first proposed by Yonekura and coworkers in 2000 [4]. In their cryo-EM study of the capped filaments, they observed the cavity at the tip of the filament enclosed by the plate region of FliD. The volume of the cavity is large enough to place no more than one partially folded FliC molecule at the time and they suggest that it can play a role of an Anfinsen cage. In the X-ray crystal structure of *Pseudomonas* FliD [8], we had observed a high degree of structural similarity between one of the head/plate domains, D3, and the D2 domain of *Pseudomonas* FliC, despite its low sequence identity. FliD could have a role in FliC chaperoning and the mechanism might derive, at least in part, from the presentation of a like-structured template in FliD against which FliC can fold. However, our experiments demonstrate that D2 and D3 domains have only a minor contribution to the FliD function and in their absence, bacteria still form fully functional flagella. Thus, the proposed chaperoning activity of FliD would rather rely on the interaction of its leg domains with the incoming FliC molecules than on the formation of an enclosed space at the tip of the filament, although a high-resolution structure of the capped filament and the knowledge on the folded state of FliC during the transport through the filament are necessary to shed light on this process.

Our results showed that D2 and D3 domains are dispensable for formation of functional filament, a surprising finding considering their apparent involvement into cap assembly [9,10,13,14]. While they may not be directly implicated in the interaction with FliC, head/plate domains may still be important in other processes, such as adhesion during infection [33,34,35,36]. Since D1 is still functional even without D2 and D3, it could be utilized to generate FliD fusion proteins with additional functions. In our experiments, we successfully introduced two small peptide tags without affecting the filament formation. Additionally, fusing D1 domain of *Pseudomonas* with significantly larger head region of *Helicobacter* FliD also yielded a functional capping structure. Surprisingly, polyhistidine loops longer than six residues do exhibit disruptive effect on a filament formation and swimming at neutral pH that can be alleviated significantly by changing the pH of the media. Imidazole group of histidine is unique among amino acid side chains in that it can be in protonated or nonprotonated state depending on the pH [37]. At pH 7, the imidazole group is nonprotonated and displays a hydrophobic character. In this case, a longer polyhistidine loop might lead to tight packing of the histidine sidechains, rendering the surrounding area more rigid, which consequently limits the movement of the D1 domain. Conversely, changing the pH of media would lead to protonation of imidazole groups, and the polyhistidine loop would unwind, allowing the helices of D1 to move more freely.

Combing all the data, we propose a model by which the interaction between FliD and FliC could occur (Figure 6D). Each FliC subunit likely moves through a series of stages of interactions and positions between its exit from the filament pore to its final resting position in the growing filament, including: (1) the “loading” step—as it exits the filament pore in its fully or partially unfolded state, each FliC subunit engages a single FliD subunit; (2) the “folding” step—the FliC subunit is transported by the FliD subunit onto which it is loaded to a position at which it can fold into its native conformation, perhaps against a like-structured region of FliD and (3) the “sorting” step—the FliC subunit is transported by the FliD subunit and placed into position in the growing filament, after which FliD disengages from FliC. Our functional, interaction and modeling data show that the coiled coil region composed of its terminal helices in the leg domain of FliD is sufficiently flexible to perform these steps. In order to perpetuate the growth of the filament, this piston-like action of the FliD coiled coil must be at different stages for each, or at least several subsets, of the FliD monomers that make up the cap assembly oligomer, depending on whether it is involved in the initial engagement, folding or ultimate placement of FliC.

## 4. Materials and Methods

### 4.1. Plasmids and Complementation of Pseudomonas aeruginosa PAO1

The coding sequences of FliD_1-474_ from the PAO1 strain of *P. aeruginosa*, FliD_1-467_ from *Salmonella typhimurium* and FliD_1-468_ from *E. coli*, optimized for expression in *P. aeruginosa* were synthesized as gene strings (Invitrogen), cloned into pBBR1MSC-5 vector, and used for *Pseudomonas* complementation experiments. Chimeric FliD genes were generated using a NEBuilder HiFi DNA assembly kit (New England Biolabs). Wild type and chimeric FliD constructs were used to transform *P. aeruginosa* PAO1 Δ*fliD* transposon strain PW2975 (obtained from the Manoil Lab at the University of Washington) by electroporation [38].

### 4.2. Swimming Motility Assays

Swimming motility assays of *Pseudomonas aeruginosa* strains were performed as described in [39]. Plates were incubated at 37 °C for approximately 20 h. The area of each bacterial swim circle was quantified using the software Image J [40]. For each complemented strain, three biological replicates with three technical replicates each were performed, and the average and standard deviations were determined. Statistical significance was determined by Brown–Forsythe and Welch ANOVA tests followed by a Dunnett’s T3 multiple comparison test using GraphPad Prism version 9.2.0 for Windows (GraphPad Software, San Diego, CA, USA).

### 4.3. Electron Microscopy

Bacteria were grown in Luria Broth liquid culture overnight at 37 °C and immobilized on a Formvar grid (Electron Microscopy Sciences, Hatfield, PA, USA) for 1 min 30 sec, and the samples were negatively stained with 0.5% (*w*/*v*) Phosphotungstic acid (PTA) and imaged using a FEI Talos 120 KV electron microscope.

### 4.4. Structural Modeling of FliD from Pseudomonas

The initial model of the full FliD protein structure was generated using the I-TASSER server [21]. Given the lack of structural templates for the N- and C-termini, and consequent low confidence assigned to these regions by I-TASSER, these regions were minimized using the FloppyTail protocol in Rosetta [24], release version 2017.29, after running FastRelax [41] in Rosetta ("relax" application) to remove any structural defects in the input model.

The FastRelax command and arguments are as follows:

relax.linuxgccrelease

- s input.pdb
- ignore_unrecognized_res
- relax:constrain_relax_to_start_coords
- relax:ramp_constraints false
- ex1
- ex2
- use_input_sc
- correct
- no_his_his_pairE
- no_optH false
- flip_HNQ
- renumber_pdb F
- overwrite
- nstruct 1

The FloppyTail command and arguments are as follows:

FloppyTail.static.linuxgccrelease

- -s input_relaxed.pdb
- -movemap movemap.txt
- -FloppyTail::shear_on 0.333
- -FloppyTail::COM_root
- -packing::repack_only
- -FloppyTail::perturb_temp 0.8
- -FloppyTail::perturb_cycles 50,000 # ~500 moves per residue
- -FloppyTail::refine_temp 0.8
- -FloppyTail::refine_cycles 1000
- -FloppyTail::refine_repack_cycles 10
- -run::min_type lbfgs_armijo_nonmonotone
- -in:file:frag3 model.500.3mers
- -nstruct 10,000

The “model.500.3mers” contains structural fragments used in the Rosetta simulation, generated using the FliD sequence, while the “movemap.txt” file specified the FliD backbone regions to move during the FloppyTail simulation. The “movemap.txt” file contents are as follows:

RESIDUE * CHI
JUMP * NO
RESIDUE 1 44 BBCHI
RESIDUE 413 474 BBCHI

The FloppyTail simulation produced 10,000 FliD models using Monte Carlo perturbation and minimization of the N-terminal and C-terminal regions. The top 200 models (2% of total) based on REF15 score, which is a recently updated version of the Rosetta energetic scoring function [42], were clustered based on pairwise backbone RMSD between models using hierarchical clustering in R (r-project.org). Three models representing observed clusters were selected. These were fit into hexameric assemblies based on the crystallographic coordinates of FliD78-405 [8] and minimized using symmetric FastRelax [41] in Rosetta to remove minor intra-subunit clashes in the hexamer, using the parameter flags noted above, in addition to parameters to enforce hexameric symmetry [43]. Ensembles of hexameric models, to identify regions of potential mobility, were generated through 100 independent trajectories of symmetric FastRelax minimization for each hexameric model, with backbone and side chain constraints (“-relax:constrain_relax_to_start_coords” and “-relax:ramp_constraints false” arguments above) omitted. Each member of the ensemble was compared with the input hexameric model to compute backbone RMSDs at each position, which were averaged across the 100 members of the ensemble.

## Data Availability

The data presented in this study are available in the Supplementary Material or upon request from M.N. or E.J.S.

## Supplementary Materials

The following are available online at https://www.mdpi.com/article/10.3390/biom11101397/s1, Supplemental Figure S1. FliD sequence alignment. ClustalOmega [44,45] sequence alignment of FliD sequences from *P. aeruginosa* PAO1 (top), *S. typhimurium* (middle) and *E. coli* (bottom) showing domain boundaries used for chimera design. Domains are colored as in Figure 1; Supplemental Figure S2. Swimming motility assays of FliD domain swaps. Representative analysis of swimming motility of FliD domain swaps. Wild type, knockout and complemented strains are shown and individually labeled. Each bacterial growth was measured as described in Materials and Methods resulting in the data shown in Figure 2; Supplemental Figure S3. Swimming motility assays of FliD helix swaps. Representative analysis of swimming motility of FliD helix swaps. Wild type, knockout and complemented strains are shown and individually labeled. Each bacterial growth was measured as described in Materials and Methods resulting in the data shown in Figure 3; Supplemental Figure S4. Swimming motility assays of FliD truncations. Representative analysis of swimming motility of FliD truncations. Wild type, knockout and complemented strains are shown and individually labeled. Each bacterial growth was measured as described in Materials and Methods resulting in the data shown in Figure 4. Supplemental Figure S5. Swimming motility assays of FliD head/plate domain replacements. Representative analysis of swimming motility of FliD head/plate domains across a pH range between 5.8 and 8.2. Wild type, knockout and complemented strains are shown and individually labeled. Each bacterial growth was measured as described in Materials and Methods resulting in the data shown in Figure 5.
