# Supplementary figures and images for: Molecular Determinants of Filament Capping Proteins Required for the Formation of Functional Flagella in Gram-Negative Bacteria
Source: Biomolecules. 2021 Sep 22;11(10):1397. doi: 10.3390/biom11101397 (PMC8533109; doi:10.3390/biom11101397)

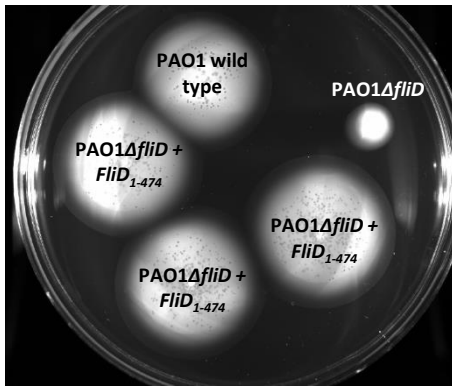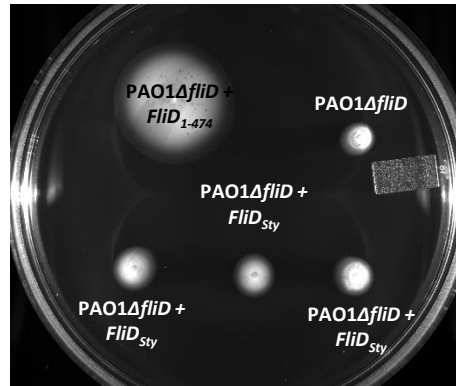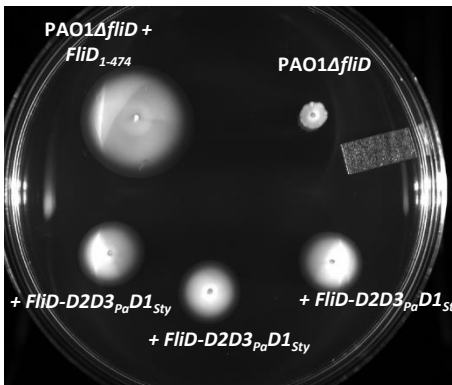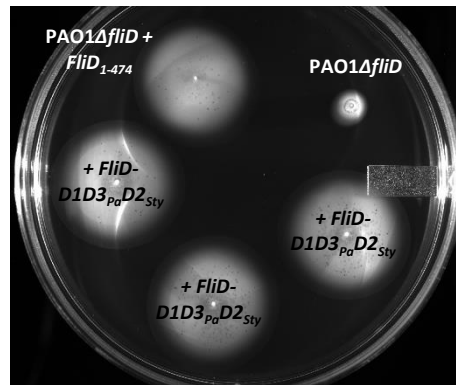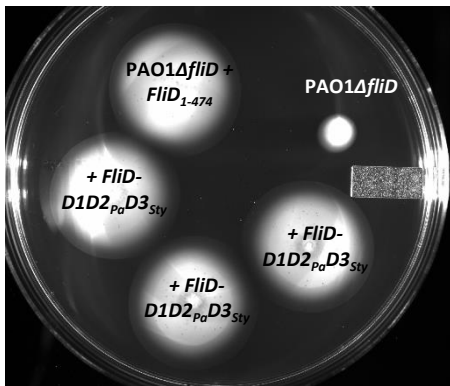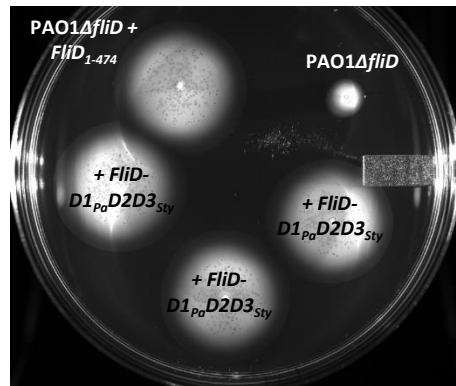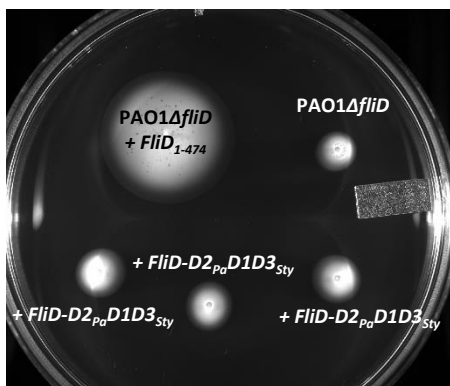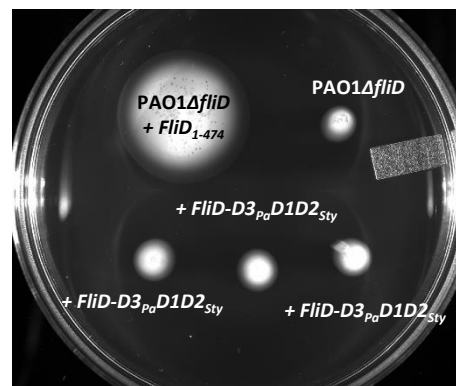

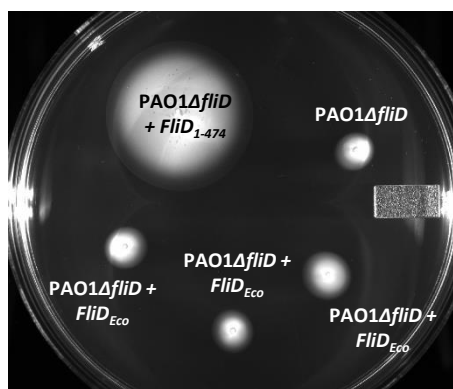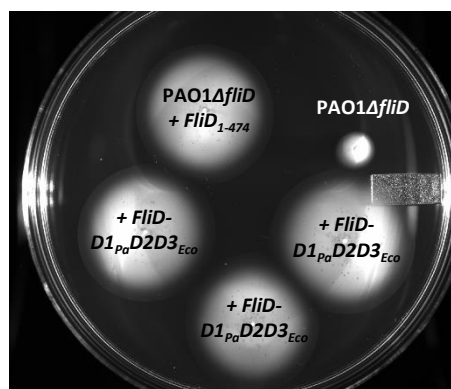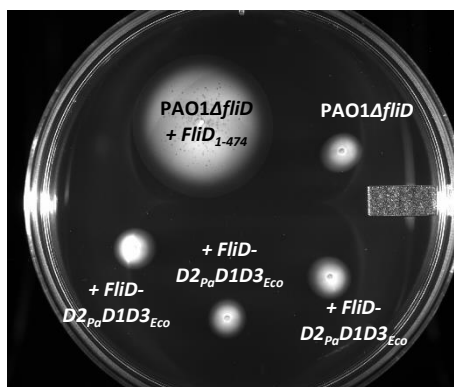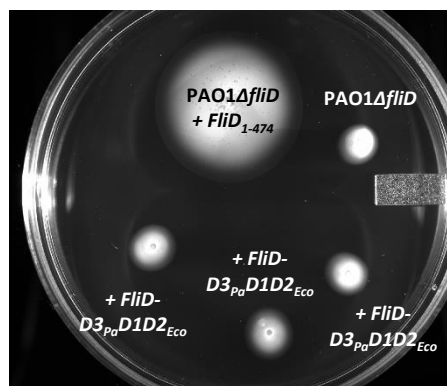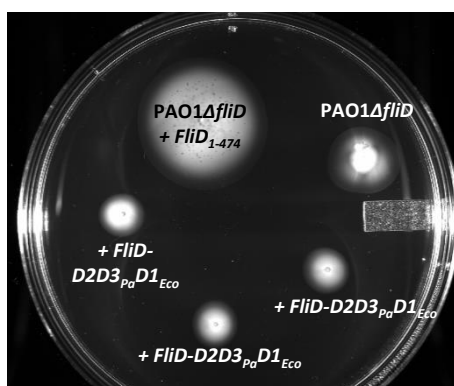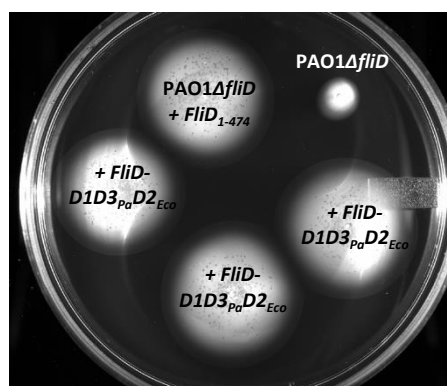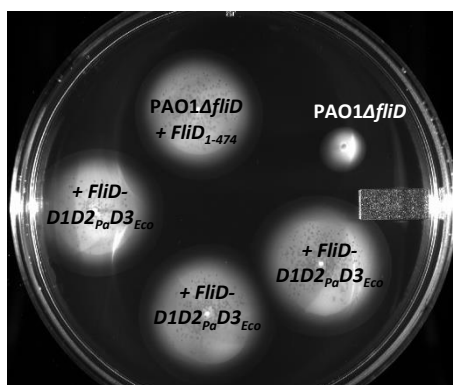

Fig. S2

Supplement: Supplementary file 1 [file biomolecules-11-01397-s001.zip › Supplementary Figure 2.pdf]

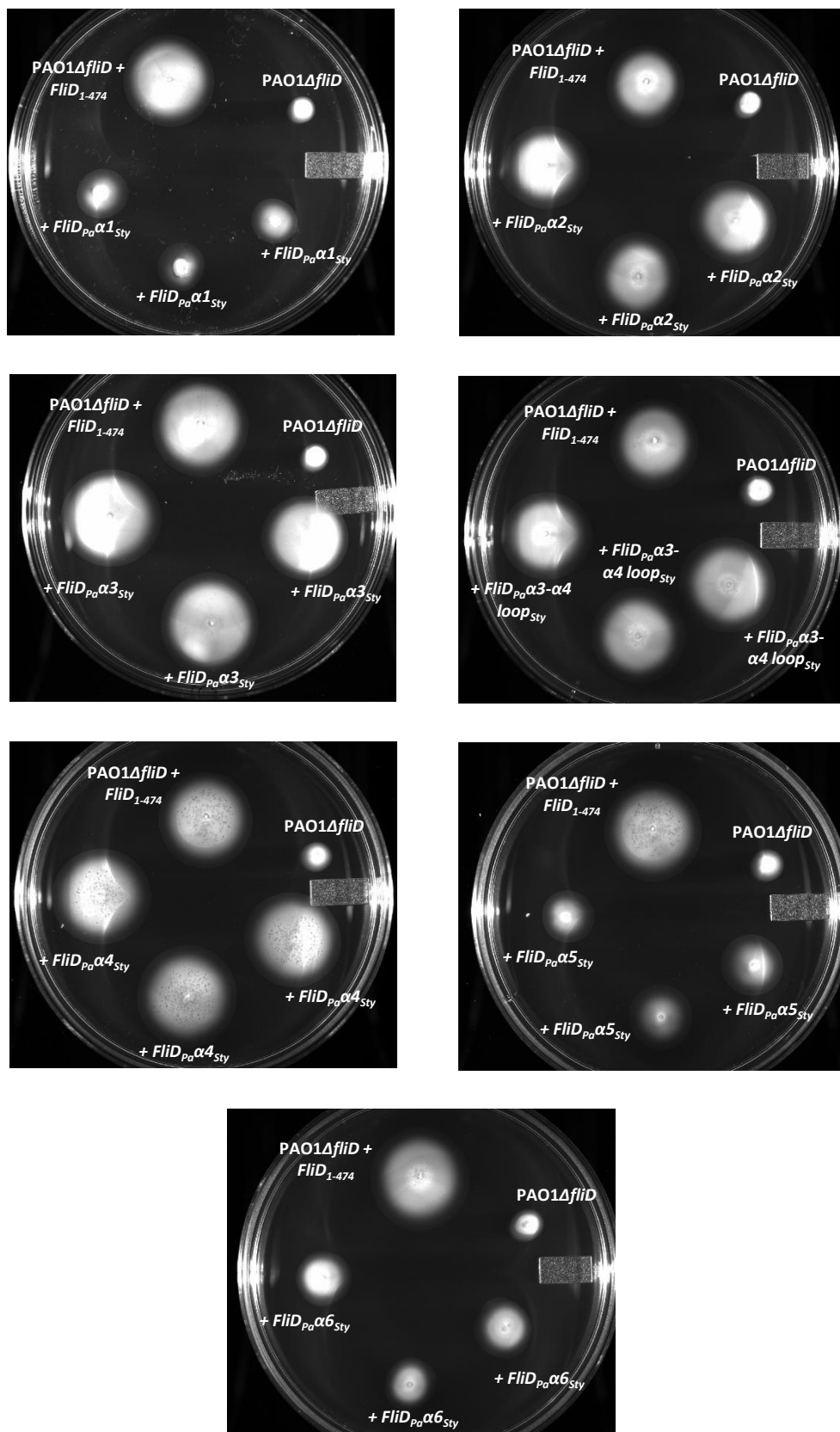

**Fig. S3**

Supplement: Supplementary file 1 [file biomolecules-11-01397-s001.zip › Supplementary Figure 3.pdf]

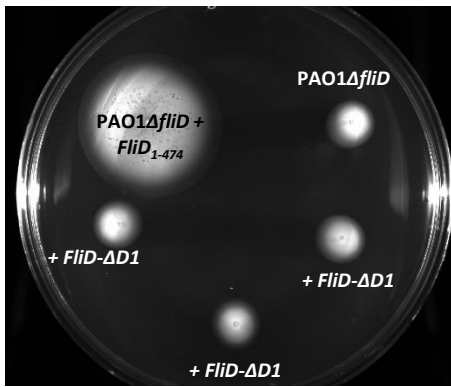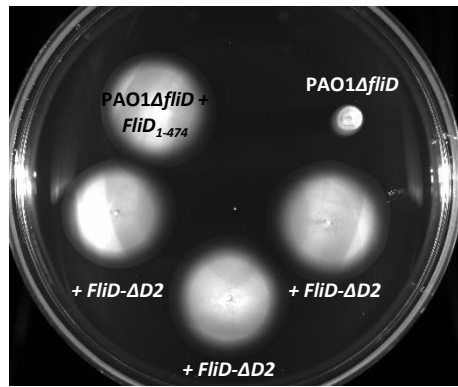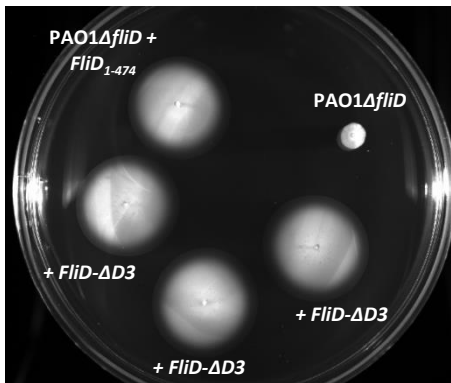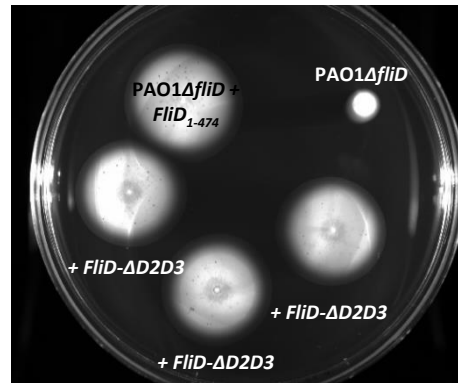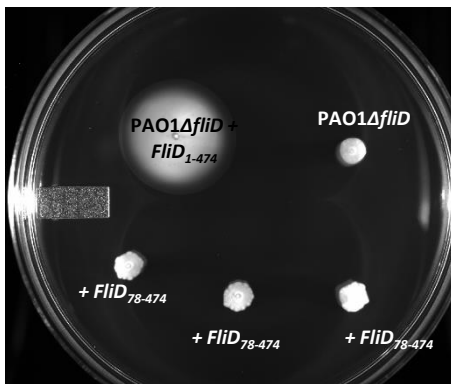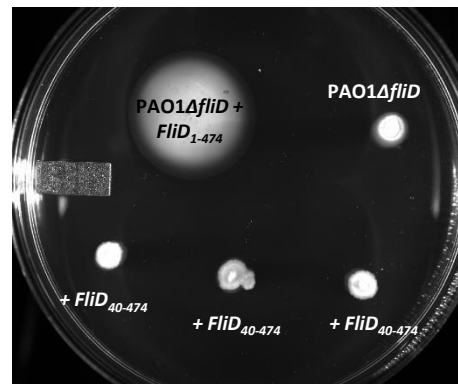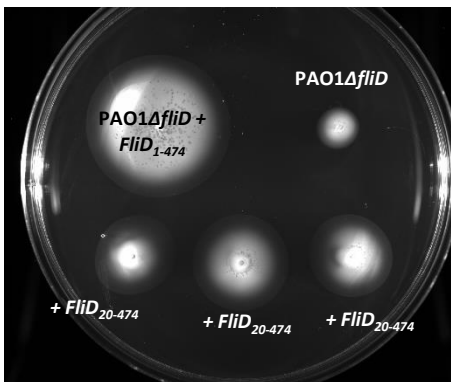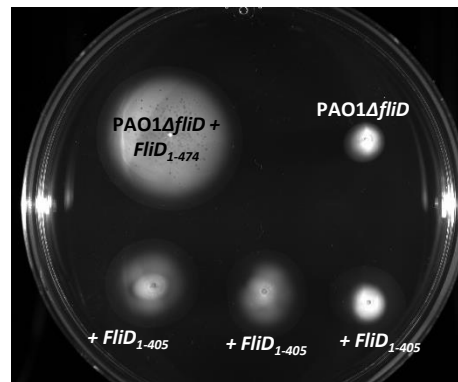

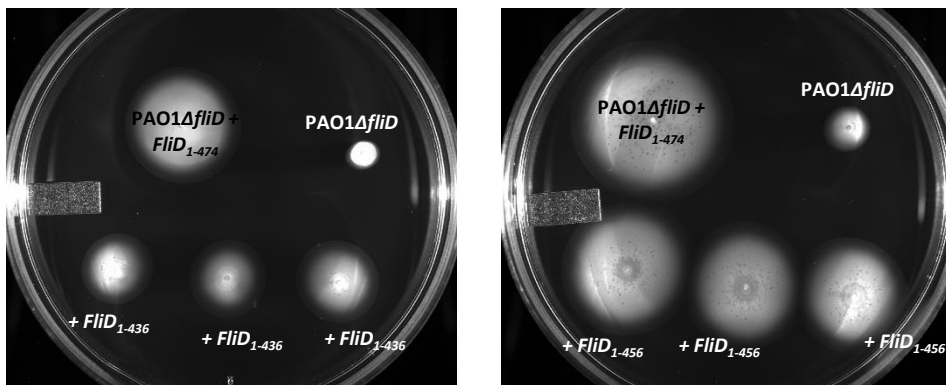

**Fig. S4**

Supplement: Supplementary file 1 [file biomolecules-11-01397-s001.zip › Supplementary Figure 4.pdf]

pH 7.0

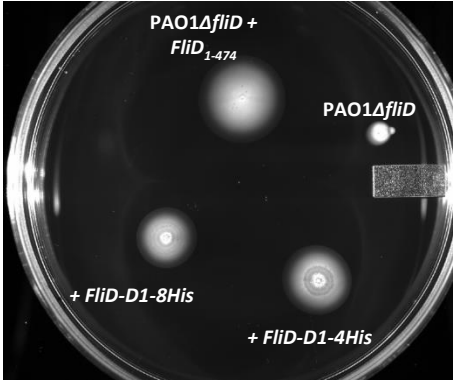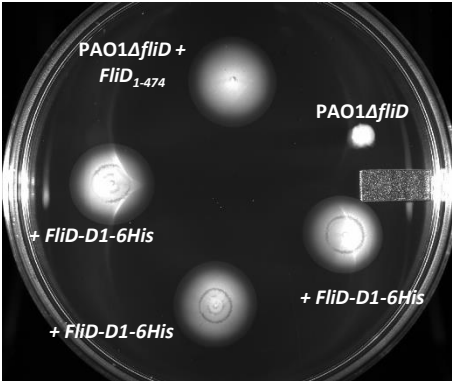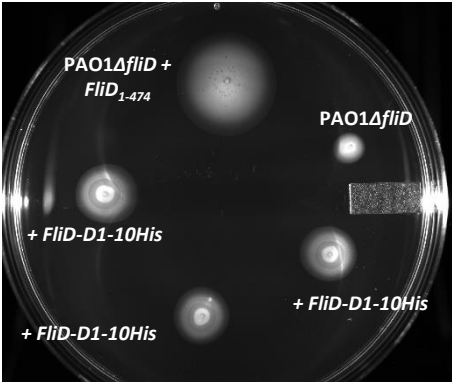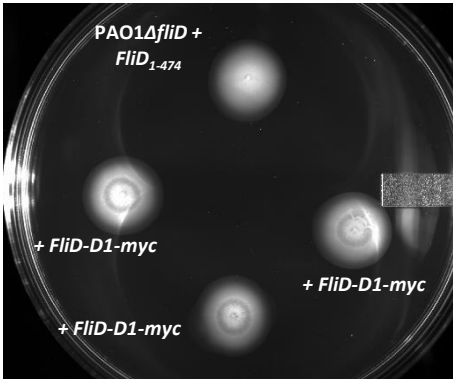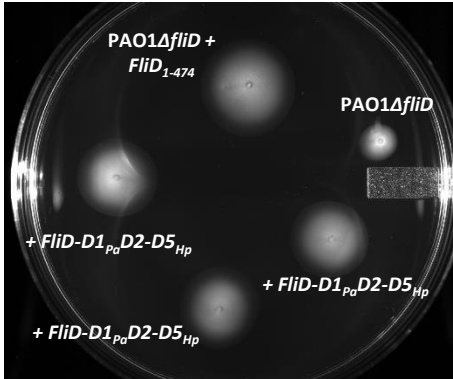

pH 5.8

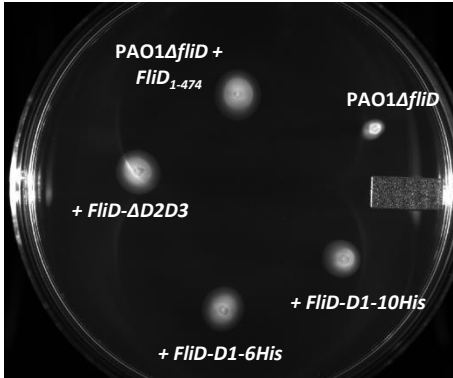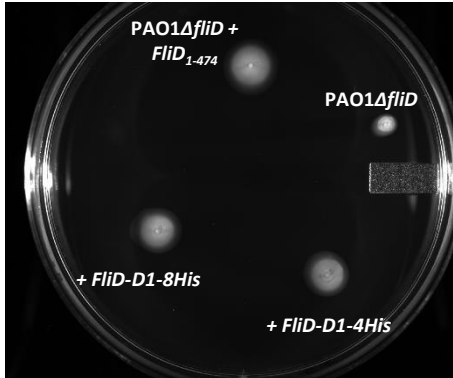

pH 6.2

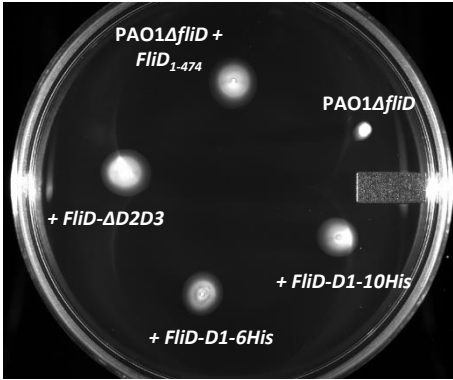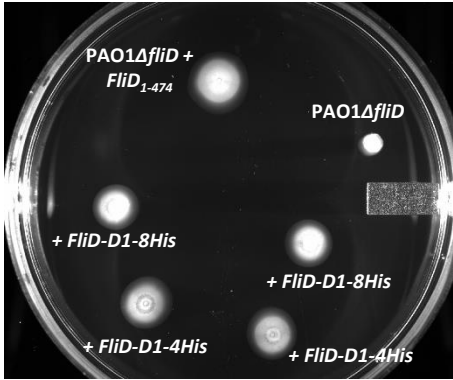

pH 6.6

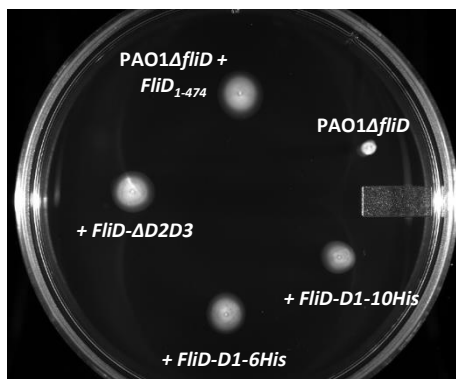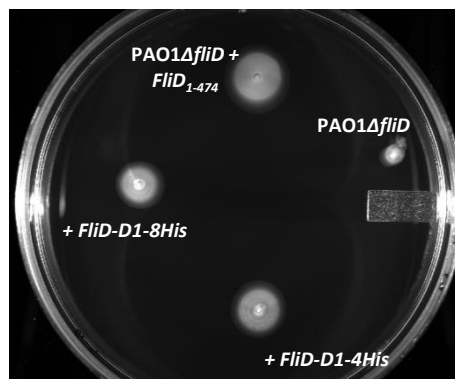

pH 7.4

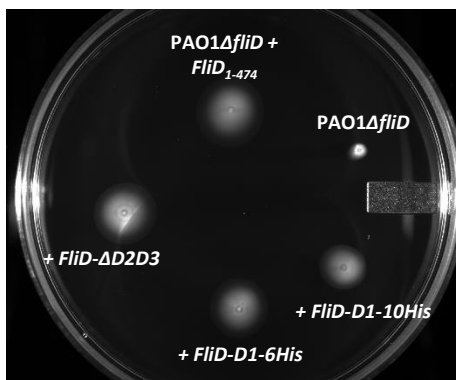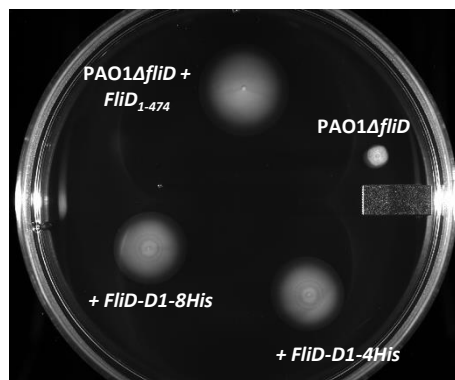

pH 7.8

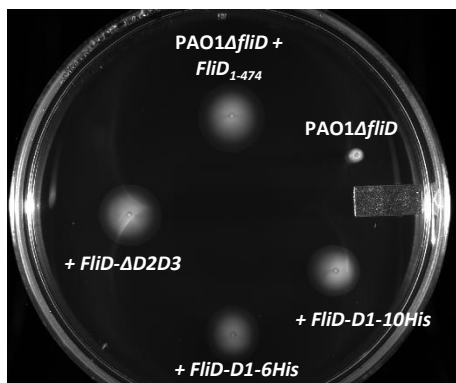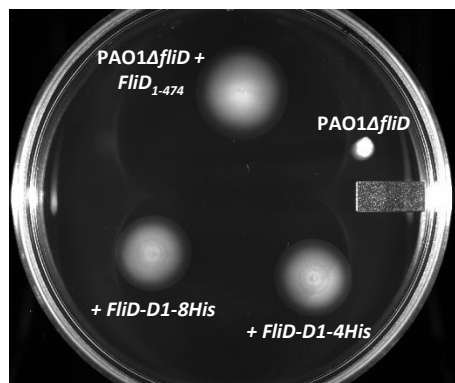

pH 8.2

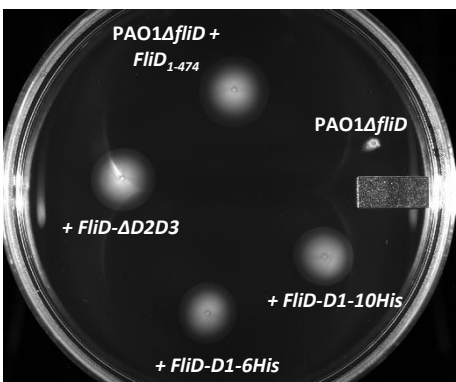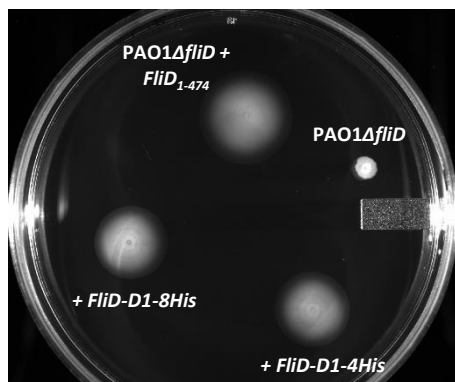

Fig. S5

Supplement: Supplementary file 1 [file biomolecules-11-01397-s001.zip › Supplementary Figure 5.pdf]
