# Supplementary material for: Molecular Determinants of Filament Capping Proteins Required for the Formation of Functional Flagella in Gram-Negative Bacteria
Source: Biomolecules. 2021 Sep 22;11(10):1397. doi: 10.3390/biom11101397 (PMC8533109; doi:10.3390/biom11101397)
Supplement: Supplementary file 1 [file Supplementary Figure 1.pdf › Supplementary Figure 1.pdf]

```
Pa        MAGI-SIGVGSTDYTDLVNKMVNLEGAAKTNQLATLEKTTTTRLTALGQFK-SAISAFQT  58
Sty       MASISSLGVGSNLPLDQLLTDLTKNEKGRLTPITKQQSANSAKLTAYGTL-KSALEKFQT  59
Ec        MASISSLGVGSGLDLSSILDSLTAAQKATLTPISNQQSSFTAKLSAYGTL-KSALTTFQT  59
          **.* *:****    . :   :.    .  . ::. :.: :::*:* * :  **:  ***

Pa        ALTALNSNAVFMARTAKSSNEDILKASATQSAVAGTYQIQVN-SLATSSKIALQAIADPA 117
Sty       ANTALNKADLFKSTVASSTTE-DLKVSTTAGAAAGTYKINVT-QLAAAQSLATKTTFATT 117
Ec        ANTALSKADLFSATSTTSSTT-AFSATTAGNAIAGKYTISVT-HLAQAQTLTTRTTRDDT 117
          * ***..  :* :  :.*:.   :..::: .* **.* *.*.* ** :..:: ::    :

Pa        NA-----KFNSGTLNISVGDTKLP-AITVDSSNNTLAGM-RDAINQAGKEAGVSATIITD 170
Sty       KEQLGDTSVTSRTIKIEQPGRKEPLEIKLDKGD-TSMEAIRDAINDAD--SGIAASIVKV 174
Ec        KTAIA---TSDSKLTIQQGGDKDPITIDISAANSSL-SGIRDAINNAK--AGVSASIINV 171
          :        .. .:.*.  . * *  * :. .: :     *****:*   :*::*:*:.

Pa        N-SGSRLVLSSTKTGDGKDIKVEVSDDGSGG-NTSLSQLAFDPATAPKLSDGAAAGYVTK 228
Sty       KENEFQLVLTA-NSGTDNTMKITVEGD-TK----LNDLLAYDSTTNT-----GNMQELVK 223
Ec        GNGEYRLSVTSNDTGLDNAMTLSVSGDDA-----LQSFMGYDASASS-----NGMEVSVA 221
            .  :* ::: .:* .: :.: *..* :  *    . :.:* ::             .

Pa        AANGEITVDGLKRSIASNSVSD-VIDGVSFDVKAVTEAGKPITLTVSRDDAGVKDNVKKF 287
Sty       AENAKLNVNGIDIERQSNTVTDAPQGI-TLTLTKKV---TDATVTVTKDDTKAKEAIKSW 279
Ec        AQNAQLTVNNVAIENSSNTISDALENITL-NLNDVT--TGNQTLTITQDTSKAQTAIKDW 278
          * *.::.*:.:  .  **:::*   .     :.  .      *:*:::* : .:  :*.:

Pa        VEAYNTLTKFINEQTV---VTKVGED-KNPVTGALLGDASVRALVNTMRSELIASNENGS 343
Sty       VDAYNSLVDTFSSLTKYTAVE-PGEE-ASDKNGALLGDSVVRTIQTGIRAQFANSGSNSA 337
Ec        VNAYNSLIDTFSSLTKYTAVDA-GADSQSSSNGALLGDSTLRTIQTQLKSMLSNTVSSSS 337
          *:***:* . :.. *    *   * :  .  .******: :*:: . ::: :  : ...:

Pa        VRNLAAL-GITTT-KDGTLEIDEKKLDKAISADFEGVASYFTGD---TGLAKRLGDKMKP 398
Sty       FKTMAEIGITQDG-TSGKLKIDDDKLTKVLKDNTAAARELLVGDGKETGITTKIATEVKS 396
Ec        YKTLAQIGITTDP-SDGKLELDADKLTAALKKDASGVGALIVGDGKKTGITTTIGSNLTS 396
           :.:* :      *..*.*::* .**  .:. :  ..   :.**   **::. :. ::.

Pa        YT-DAQGILDQRTTTLQKTLSNVDTQKADLAKRLAALQEKLTTQFNLLSAMQD-EMTKRQ 456
Sty       YLAD-DGIIDNAQDNVNATLKSLTKQYLSVSNSIDETVARYKAQFTQLDTMMSKL-NNTS 454
Ec        WLST-TGIIKAATDGVSKTLNKLTKDYNAASDRIDAQVARYKEQFTQLDVLMTSL-NSTS 454
          :     **:.     :. **..: .:    :. :     : . **. *..:     .. .

Pa        KSITDNLASLPYGSGKKT   474
Sty       SYLTQQFTAMNKS-----   467
Ec        SYLTQQFENNSNSK----   468
          . :*:::     .
```

- D1 α1
- D1 α2
- D2
- D3
- D1 α3
- D1 α3-α4 loop
- D1 α4
- D1 α5
- D1 α6

Fig. S1
